# *In vitro* and *in silico* assessment of new beta amino ketones with antiplasmodial activity

**DOI:** 10.1590/0037-8682-0590-2022

**Published:** 2022-09-26

**Authors:** Gabriela Camila Krombauer, Karla de Sena Guedes, Felipe Fingir Banfi, Renata Rachide Nunes, Amanda Luisa da Fonseca, Ezequias Pessoa de Siqueira, Jéssica Côrrea Bezerra Bellei, Kézia Katiani Gorza Scopel, Fernando de Pilla Varotti, Bruno Antônio Marinho Sanchez

**Affiliations:** 1 Universidade Federal de Mato Grosso, Núcleo de Pesquisa e Apoio Didático em Saúde, Laboratório de Imunopatologia e Doenças Tropicais, Sinop, MT, Brasil.; 2 Universidade Federal de São João Del Rei, Campus Centro Oeste, Núcleo de Pesquisa em Química Biológica (NQBio), Divinópolis, MG, Brasil.; 3 Fundação Oswaldo Cruz, Instituto René Rachou, Laboratório de Química, Belo Horizonte, MG, Brasil.; 4 Universidade Federal de Juiz de Fora, Centro de Pesquisas em Parasitologia, Departamento de Parasitologia, Microbiologia e Imunologia, Juiz de Fora, MG, Brasil.

**Keywords:** Malaria, Chemotherapy, Antimalarial, Docking

## Abstract

**Background:**

Based on the current need for new drugs against malaria, our study evaluated eight beta amino ketones *in silico* and *in vitro* for potential antimalarial activity.

**Methods:**

Using the Brazilian Malaria Molecular Targets (BraMMT) and OCTOPUS® software programs, the pattern of interactions of beta-amino ketones was described against different proteins of *P. falciparum* and screened to evaluate their physicochemical properties. *The in vitro* antiplasmodial activities of the compounds were evaluated using a SYBR Green-based assay. In parallel, *in vitro* cytotoxic data were obtained using the MTT assay.

**Results:**

Among the eight compounds, compound 1 was the most active and selective against *P. falciparum* (IC_50_ = 0.98 µM; SI > 60). Six targets were identified in BraMMT that interact with compounds exhibiting a stronger binding energy than the crystallographic ligand: *P. falciparum* triophosphate phosphoglycolate complex (1LYX), *P. falciparum* reductase (2OK8), PfPK7 (2PML), *P. falciparum* glutaredoxin (4N0Z), PfATP6, and PfHT.

**Conclusions:**

The physicochemical properties of compound 1 were compatible with the set of criteria established by the Lipinski rule and demonstrated its potential as a drug prototype for antiplasmodial activity.

## INTRODUCTION

Malaria continues to be one of the most important public health problems, with an estimated > 400,000 deaths each year^1^ ^,^ ^2^. In humans, the disease is caused by the protozoan species of the *Plasmodium* genus^3^. 

Although several substances are used in antimalarial chemotherapy, many of them are no longer used for treatment because of their side effects or the development of parasitic resistance^4^ ^-^ ^11^. Therefore, new strategies should be used, such as the addition of a third drug with independent antiparasitic activity^10^.

Computational tools have been employed to understand complex interactions in biological models^12^. 

The main objective of the computational model is to replicate the patterns of biological systems^13^ with a high accuracy. *In silico* models that achieve this goal are an important complement to experimental studies and can provide valuable insights into the mode of action of bioactive compounds^14^. 

In this context, virtual screening (VS) is an important methodology in the discovery process of new antimalarial candidates^15^ and enables the identification of potential targets, contributing to the elucidation of its mechanism of action^16^. 

Heterocyclic compounds are potential pharmacotherapeutic agents, which include antihypertensive effects and antimicrobials^17^ ^-^ ^20^. Morpholine, piperidine, and their derivatives are examples of heterocyclic compounds that contain a nitrogen atom^21^ ^-^ ^31^. In the specific context of malaria, piperidine derivatives are low-cost synthesized compounds with efficient antimalarial activity^32^. In addition, morpholine scaffolds have already been proven to be good starting points for the development of new antimalarial candidates^33^. 

Thus, VS associated with biological assays is an increasingly useful approach for developing tools to identify potential antimalarial scaffolds^34^. Therefore, the present study aimed to evaluate the antiplasmodial activities of eight compounds synthesized from beta-amine ketones *in silico* and *in vitro*.

## METHODS

### Synthesis, Purification and Structural elucidation

**FIGURE 1:**
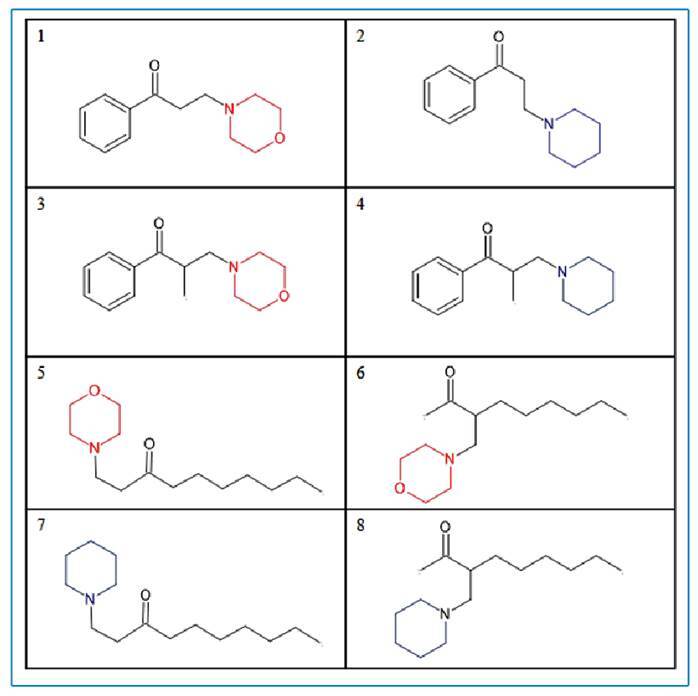
Molecular structures of compounds with morpholine (1-4) and piperidin (5-8).

Eight molecules were obtained: morpholines (1, 3, 5, and 6) and piperidines (2, 4, 7, and 8) (Figure 1). A description is provided in the supplementary material.

### Antiplasmodial activity

*P. falciparum* strain W2 (chloroquine-resistant) was cultured as previously described^36^ ^,^ ^37^. The antiplasmodial activity of ß-amino-cetones (compounds 1-8) against *P. falciparum* cultures was evaluated using SYBR^38^. Ring-stage parasites were equally distributed in 96-well microculture plates. Serial dilutions of the compounds were performed ranging from 0.01 to 100 μg/mL. Chloroquine (CQ) was used as the antimalarial control. A fluorometer (Fluoroskan Ascent, Thermo Laboratories) with excitation at 485 nm and emission at 535 mm was used to determine the viability of the parasites. All experiments were performed in triplicates. Results are expressed as the mean of the IC_50_ (drug concentration that reduced parasite viability by 50%).

### *In vitro* cytotoxicity

All compounds were assessed against WI-26VA4 (ATCC CCL-95.1, USA) human pulmonary fibroblast cells by MTT assay^39^. Cells were cultured in RPMI-1640 medium (Sigma-Aldrich ^®^, St. Louis, Missouri, USA) supplemented with 10% fetal bovine serum in 96-well plates^40^. Compounds 1-8 were diluted to concentrations ranging from 0.2-200 μg/mL and incubated for 48 h in a 5% CO_2_ atmosphere at 37 ºC.

Cellular viability was determined at 540 nm to measure the signal and background (Spectra Max340PC, Molecular Devices, Sunnyvale, California, USA). The minimum lethal dose for 50% of the cells (LD_50_) was determined as previously described^41^.

### Selectivity index (SI)

Selectivity index (SI) is the ratio between the values of LD_50_ and IC_50_ cytotoxicity of each compound tested. Values greater than 10 were considered low cytotoxic, whereas values below 10 were considered cytotoxic^42^. 

### Statistical analysis

IC_50_ and LD_50_ were determined using the equation of the curve obtained by plotting parasitemia reduction (%) or cellular death (%) vs. the concentration of the compound (log scale) using the GraphPad Prism software (version 5.0 for Windows, San Diego, California, USA). Due to the non-normality of the data distribution, the comparison of IC_50_ and LD_50_ between morpholine and piperidine was analyzed using the nonparametric Mann-Whitney U test. Statistical significance was defined at 5% (p<0.05).

### Evaluation of virtual screening and ADMET properties

Description is available in the supplementary material.

## RESULTS

### Antiplasmodial activity

The antiplasmodial activities of these eight compounds are listed in Table 1. IC_50_ values in the *in vitro* antimalarial tests (W2 strain) ranged from 0.98 μM to 47.95 μM. Compounds 1 and 4 had IC_50_ values ​​close to those of chloroquine, a standard antimalarial drug. There was no statistically significant difference in IC_50_ or LD_50_ between parasites treated with morpholine and piperidine (p = 0.225 and p = 0.593, respectively).

**TABLE 1:** Morpholines and piperidines antiplasmodial activity.

Compounds	IC_50_ ± SD (μM)^*^	LD_50_^a^ ± SD (μM)^*^	SI
**Morpholines**			
1	0.98 ± 0.01	59.78 ± 6.83	61
3	32.53 ± 0.02	>100	3.07
5	3.93 ± 0.01	80.32 ± 9.03	20.44
6	3.93± 0.02	94.45 ± 18.90	24.03
Piperidines			
2	3.59± 0.08	33.97 ± 18.91	9.46
4	0.78± 0.09	>100	128.21
7	2.49± 0.03	>100	40.16
8	47.95 ± 18.51	38.82 ± 1.16	0.81
Chloroquine (CQ)	0.92 ± 0.01	>100	108.70

Drug concentration that reduced parasite viability in 50% (IC_50_) and WI-26VA4 cells viability in 50% (LD_50_), and selectivity index (SI) values of the compounds.

*Mean and standard deviation (SD) of triplicate experiments. The test of the hypothesis of equality of IC_50_ and LD_50_ between the morpholine and piperidine groups was p=0.225 and 0.593, respectively (Mann-Whitney U test).

### Cytotoxic activity

The compounds did not show cytotoxic activity, since the LD_50_ values ranged from 33.97 μM to >100 μM compared to the control drug (chloroquine) (Table 1). 

Compounds 2, 3, and 8 were not selective, with SI values below 10 (9.46, 3.07, and 0.81, respectively). Compounds 1, 4, 5, 6, and 7 showed the highest selectivity (SI>10), with values ranging from 20.44 to 128.21 (Table 1).

Compound 1 stood out for its IC_50_ corresponding to 0.98, as well as for its SI of 61 and the values of binding energy it exhibited against the targets presented in BraMMT (Brazilian Malaria Molecular Targets), as described below.

Therefore, the docking assay and physicochemical properties of this compound were analyzed.

### Virtual screening

Table 2 presents the molecular targets, locations, and enzymatic classes of the 35 proteins listed in the Brazilian Malaria Molecular Targets (BraMMT). Virtual screening of the compounds was performed against all 35 BraMMT.

**TABLE 2:** *Brazilian Malaria Molecular Targets* (BraMMT).

PDB Code	Name	Enzymatic class	Location
1LF3	Plasmepsin II	Hydrolase	Digestive vacuole
1LYX	Triosephosphate Isomerase (PfTIM)-Phosphoglycolate	Isomerase	Cytoplasm
1NHW	Enoyl-acyl-carrier-protein reductase	Oxidoreductase	Apicoplast
1O5X	Triosephosphate Isomerase	Isomerase	Cytoplasm
1QNG	Peptidyl-prolil cis-trans isomerase	Isomerase	Cytoplasm
1RL4	Formylmethionine deformylase	Hydrolase	Apicoplast
1TV5	Dihydroorotate dehydrogenase	Oxidoreductase	Cytoplasm e Nucleus
1U4O	L-lactate dehydrogenase	Oxidoreductase	Cytoplasm
1YWG	glyceraldehyde-3-phosphate dehydrogenase	Oxidoreductase	Cytoplasm
2AAW	Glutathione s-transferase	Transferase	Cytoplasm
2ANL	Plasmepsin IV	Hidrolase	Digestive vacuole
2OK8	Putative ferredoxin--NADP reductase	Oxidoreductase	Apicoplast
2PML	Ser/Thr protein kinase	Transferase	Cytoplasm
2Q8Z	Orotidine-monophosphate-descarboxylase	Liase	Nucleus
2VFA	Hypoxantine-guanine phosphoribosyltransferase	Transferase	Apicoplast
2VN1	70 KDA peptidylprolyl isomerase	Isomerase	Nucleus
2YOG	Thymidylate kinase	Transferase	Nucleus
3AZB	Beta-hydroxyacyl-ACP dehydratase	Lyase	Cytoplasm
3BPF	Falcipain II	Hydrolase	Digestive vacuole
3CLV	Rab5 Protein	Signaling protein	Cytoplasm
3FNU	HAP Protein	Hydrolase	Digestive vacuole
3K7Y	Aspartate aminotransferase	Transferase	Cytoplasm
3N3M	Orotidine 5'-phosphate decarboxylase	Lyase	Apicoplast
3PHC	Purine nucleoside phosphorylase	Transferase	Nucleus
3QS1	Plasmepsin I	Hydrolase	Digestive vacuole
3T64	Deoxyuridine 5'-triphosphate nucleotidohydrolase	Hydrolase	Nucleus
3TLX	Adenylate kinase 2	Transferase	Cytoplasm and mitochondria
4B1B	Thioredoxin reductase	Oxidoreductase	Cytoplasm
4C81	22-C-Methyl-D-Erythritol 2,4-Cyclodiphosphate synthase	Lyase	Apicoplast
4J56	Thioredoxin reductase 2	Oxidoreductase	Cytoplasm
4N0Z	Glutaredoxin	Oxidoreductase	Cytoplasm
4P7S	Macrophage migration inhibitory factor-like protein	Cytokine inhibitor	Cytoplasm
4QOX	Calcium-dependent protein kinase 4	Transferase	Cytoplasm
PfATP6	Calcium pump ortholog ATPase	Transporter	Membrane
PfHT (10.5452/ma-aej21)	Hexose carrier protein	Transporter	Membrane

As shown in Table 3, six of the 35 targets that make up BraMMT interacted with compounds 1-8, presenting binding energies superior to the crystallographic data. The six targets that were linked to the compounds were: *P. falciparum* triosphosphate phosphoglycolate complex (1LYX), ferredoxin-NADP + *P. falciparum* reductase (2OK8), crystal structure of PfPK7 (2PML), oxireduction protein of *P. falciparum* glutaredoxin (4N0Z), *P. falciparum* ATPase orthologous calcium pump (PfATP6), and *P. falciparum* hexose transporter (PfHT). Compound 4 stood out on presenting the most satisfactory result, establishing a connection with the six targets, and presenting a higher binding value than the crystallographic data. 

**TABLE 3:** Binding energies of compounds against BRAMMT targets (kcal.mol^-1^).

Compounds	Molecular targets					
	Binding energy (kcal.mol^-1^)					
	1LYX	2OK8	2PML	4N0Z	PfATP6	PfHT
1	-5.9	-4.4	-6.6	-4.9	-6.3	-6.5
3	-5.5	-4.5	-7.0	-4.9	-6.8	-6.3
5	-5.9	-3.6	-5.9	-4.1	-5.5	-5.6
6	-5.5	-3.7	-6.2	-4.3	-6.0	-5.9
2	-5.8	-4.4	-7.1	-4.6	-6.8	-6.7
4	-5.8	-4.6	-6.9	-4.7	-7.3	-6.5
7	-5.8	-3.7	-5.8	-4.2	-6.1	-5.5
8	-4.8	-3.7	-6.3	-4.3	-6.1	-6.1
Crystallographic	-5.6	-2.0	-6.9	-4.3	-7.2	-5.7

All tested compounds (1-8) interacted with the 2OK8 target, in which they obtained a stronger binding energy with the target than the crystallographic ligand. Compounds 1, 2, 3, 4, 6, and 8 interacted with the 4N0Z target. Compound 4 interacted with PfATP6, obtaining a similar value of binding energy (-7.3 kcal/mol) with the target compared to the crystallographic ligand (-7.2 kcal/mol). 

Compounds 1 (-6.5 kcal/mol), 2 (-6.7 kcal/mol), 3 (-6.3 kcal/mol), 4(-6.5 kcal/mol), 6 (-5.9 kcal/mol), and 8 (-6.1 kcal/mol) obtained stronger binding energy with the target than the crystallographic ligand (-5.7 kcal/mol), demonstrating interaction with PfHT. Furthermore, compound 1 stood out in the antimalarial and cytotoxicity tests and was therefore chosen for intermolecular and physicochemical analyses. 

**FIGURE 2:**
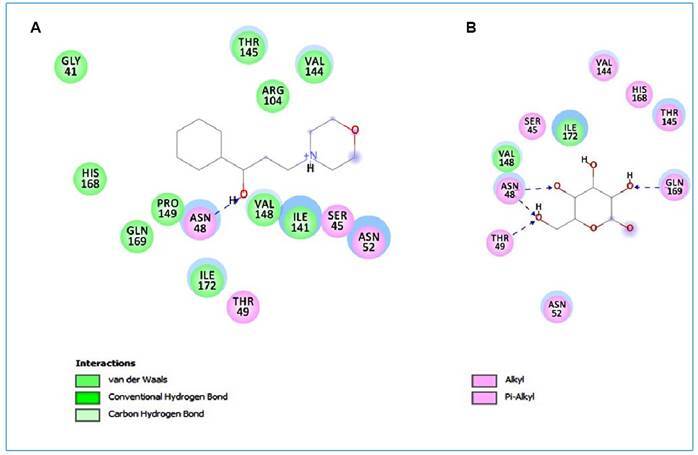
Intermolecular interactions with PfHT. **A:** Compound 1; **B:** D-glucose.

Figure 2 shows a two-dimensional map of linker-receptor interactions with PfHT and the chemical bonds between compound 1 and the target. Pharmacophoric groups and possible structural improvements in permeability, absorption, and oral bioavailability have been indicated. By analyzing the molecular interactions of 1 with PfHT, it was possible to observe the interaction of 1 with GLN169 and THR145 residues (Figure 2). Molecular anchoring with D-glucose was performed to recognize the interactions at the PfHT binding site.

### Tests of physicochemical properties

Supplementary Table 1 shows SwissADME profiles of compound 1 and chloroquine. Compound 1 had a CLogP <5 (1.67) and a molecular weight less than 500 g/mol (219.28 g/mol) (Supplementary Table 1). There were three hydrogen acceptor groups that performed the interactions and no hydrogen donor groups.

Compounds with CLogP values less than 3 present a low risk of side effects and toxicity, which indicates a low risk of retention and storage of the compound. Patterns of mutagenicity, tumorigenicity, and irritability were not associated with the molecular structure of compound 1, corroborating the literature, since this compound had CLogP <3 (Supplementary Table 1). 

In addition, compound 1 had a superior synthetic facility compared to chloroquine, standing out for its solubility in water.

## DISCUSSION

The committee coordinated by the Global Health Innovative Technology (GHIT) foundation, guided by the specific requirements for the disease and considering the target product and candidate profiles, defined a set of criteria in an attempt to validate new antimalarial candidates. These criteria were divided into validation and activity/mechanism of action, LD_50_ values for cell lines, IC_50_ of the parasite, and selectivity index. The compounds must be tested according to the study of the life cycle of *Plasmodium* to evaluate their mechanism of action, correlating the manifestations of malaria. The main tests must be performed according to the target to ensure the characterization of the potential target candidate^49^.

The *in vitro* cultivation of *P. falciparum* strains is the main test model for screening new compounds and monitoring resistance to antimalarial drugs, since it is possible to observe the activity, dosage, and resistance of natural and synthetic compounds through these tests^50^.

IC_50_ values must be <1 μM to validate compounds or effective compounds for *Plasmodium spp*. The selectivity index must be greater than 10 (LD_50_ for the cell line relative to the IC_50_ of the parasite) for *Plasmodium spp* ^49^. Thus, compound 1 meets two parameters with IC_50_ < 0.98 µM and IS=61. Compounds 4, 5, 6, and 7 presented selectivity indices of 128, 20.44, 24, and 40.16, respectively, which are satisfactory; however, the IC_50_ was higher than 1 μM.

A study evaluating the antimalarial activity of alkaloids isolated from *Aspidosperma ulei* marker demonstrated IC_50_ values close to 20 μM, indicating moderate activity against chloroquine-resistant *P. falciparum* strains^51^. According to these parameters, compounds 1 (0.98 μM), 2 (<3.59 μM), 5 (3.93 μM), and 6 (<3.93) were considered potentially active.

In a study by Ohashi^52^, it was demonstrated that the potential activity of a given compound varies between the protozoan species, obtaining different SI values according to the species analyzed. These results demonstrate that it is essential to understand the mechanisms of action of the compounds and cell lines used in the cell viability tests, in addition to the activity of the analyzed substances. Compound 1 stood out for its IC_50_ of 0.98, as well as an IS of 61. Thus, the physicochemical properties of this compound were analyzed.

Virtual screening of substances derived from the Mannich reaction was performed using 35 molecular targets obtained from BRAMMT. As shown in Table 3, 1LYX, 2OK8, 2PML, 4N0Z, PfATP6, and PfHT targets presented the best binding energy values for the compounds.

Using the BraMMT tool, the identification of a potential target is crucial in the discovery and rational development of new drugs to show their biological importance concurrently with the validation of the methodology to be used^53^. Each *P. falciparum* target present in the bank was used to perform virtual screening and was identified according to its registration in the Protein Data Bank. 

Ferredoxin-NADP + reductase from *P. falciparum* (PDB code: 2OK8) is associated with apicoplast metabolic pathways, a key organelle for parasite survival. Thus, *P. falciparum* exhibits a ferredoxin-NADP + reductase, giving rise to reduced ferredoxin for the essential biosynthetic pathways of the apicoplast^54^. All tested substances (1-8) obtained binding energies stronger than the crystallographic ligand with the 2OK8 target.

*P. falciparum* glutaredoxin 1 (PDB 4N0Z) is an oxireduction protein present in the cytoplasm of the parasite that uses glutathione as a cofactor (glutaredoxin). This protein is exclusively found in *Plasmodium* species and plays a role in the central performance of maintaining parasite homeostasis^55^. Compounds 1, 2, 3, 4, 6, and 8q exhibited stronger binding energies with the 4N0Z target than with the crystallographic ligand.

*P. falciparum* ATPase orthologous calcium pump (PDB PfATP6) is present in the sarcoplasmic reticulum of *P. falciparum*. The cytoplasmic calcium concentration increases with inhibition and therefore compromises the parasite’s signaling pathways^56^. Compound 4 obtained a higher binding energy value with the target than that of the crystallographic ligand, presenting a binding energy of -73 kcal.mol^-1^ compared to the value of -72 kcal.mol^-1^ of the crystallographic ligand, demonstrating an interaction with PfATP6.

The hexose transporter of *P. falciparum* (PDB PfHT) is a transmembrane protein responsible for glucose transport. Plasmodium requires glucose as its primary energy source during the erythrocytic cycle. Inhibition of glucose transport to infected cells impairs parasite viability. Therefore, substances that inhibit PfHT can be considered promising templates for developing new antimalarial compounds^57^ ^,^ ^58^ ^,^ ^59^. 

Compounds 1, 2, 3, 4, 6, and 8 exhibited stronger binding energies with PfHT than with the crystallographic ligand.

The *P. falciparum* Triosphosphate Isomerase Phosphoglycolate complex (PDB 1LYX) was also identified as *a P. falciparum* Triosephosphate isomerase (PfTIM). This consists of a catalytically active enzyme, which is the subject of many studies because of its particularity at position 96. There is a serine residue in region 96 of triosphosphate isomerase in both humans and other organisms. However, the residue in PfTIM was replaced by a phenylalanine residue, with this enzyme being the only one to have serine in that position; however, so far, no specific role has been attributed to this particularity^60^ ^,^ ^61^.

Differences were found when comparing the PfTIM structure with other TIM structures, especially that of humans, which can assist in modeling and developing therapeutic targets. One of these differences and particularities of PfTIM is in position 183, which has a completely exposed leucine, different from TIMs of other organisms, which have glutamate. It is believed that this exposed leucine residue, together with the positively charged surrounding adhesive, may be responsible for TIM binding to the erythrocyte membrane, which is important for the energy production of *Plasmodium* ^62^. 

The crystalline structure of PfPK7 in complex with an ATP analog (PDB 2PML) is a protein kinase that plays an important role in regulating the development of the parasite, by being involved in the signaling pathway of melatonin and modulating the life cycle of *P. falciparum* ^63^ ^,^ ^64^ *.* PfPK7 is expressed in both the asexual and sexual stages of the parasite.

Dorin-Semblat et al.^65^ demonstrated that interruption of the PfPK7 gene resulted in a reduction in the number of merozoites produced by each schizont, and the ability to produce oocysts in the mosquito was also impaired. These data reinforce the importance of PfPK7 in the parasite’s life cycle^63^. Several molecular targets, from subcellular organelles to metabolic pathways, have been described in the literature in an attempt to fight infection^66^ ^,^ ^67^. Despite the numerous potential targets of pharmacological action, the most used compounds still date back many years: quinine, isolated from the *Cinchona sp.* in 1820 and artemisinin, purified from *Artemisia annua* in 1972, both extracted from natural sources^68^. 

*P. falciparum* hexose transporter (PfHT) has recently been characterized as promising for the development of new drugs^59^ ^,^ ^60^ ^,^ ^69^. The reason for this is that the parasite is found inside erythrocytes surrounded by the parasitophore vacuole, and glucose molecules must pass through its membrane before being transported to the parasite^70^ ^,^ ^71^. Therefore, PfHT was characterized among BraMMT with similar or superior binding energy to the crystallographic ligand as a carrier target, which can contribute to the performance of the prototype compounds.

Compound 1 was chosen for the analysis of its physicochemical properties and intermolecular interactions according to its performance in antimalarial and toxicity tests. Compound 1 interacts with GLN169 and THR145 residues at the same D-glucose binding site in PfHT^59^.

Figure 2 shows the electrostatic bonds and Van der Waals interactions with the PfHT receptor by the GLY41, SER45, ASN48, THR49, ASN52, ARG104, ILE141, VAL144, THR145, VAL148, PRO149, HIS168, GLN169, and ILE172 groups and the SER45, ASN48, ASN52 THR49, VAL144, THR145, VAL148, HIS168, GLN169, and ILE172 nuclei with D-glucose, indicating that the antimalarial activity is associated with the presence of these structures.

The hexose transporter for *P. falciparum* (PfHT) was recently described by molecular anchoring, QM/MM, and simulations of molecular dynamics by our research group^59^ ^,^ ^60^. Figure 2 shows a two-dimensional map of ligand-receptor interactions with PfHT. The chemical bonds between the compound and the target are indicated. The two-dimensional structure of these compounds is also shown. Molecular anchoring with D-Glucose was performed to recognize the interactions at the PfHT binding site.

Identifying and characterizing pharmacologically active molecules is a necessity that has led to efforts in diverse areas of scientific research worldwide. The applicability of such molecules can be quite variable; however, the main objective of the drug development process is to obtain molecules with efficiency and specificity in relation to the therapeutic target, low cost, and absence or reduction of risks to potential users^72^. 

The increase in resistance to current antimalarial drugs drives research that aims to understand the mechanism of action of compounds for parasitic diseases with social and economic impacts. This knowledge will assist in screening bioactive compounds with determined pharmacodynamic and/or pharmacokinetic characteristics^73^. The methodologies used in this study have the main objective of characterizing potential targets, and these data and discussions allow us to infer the importance of the present study.

PfHT has characteristics similar to those of the human glucose transporter (GLUT1). However, there are some differences in their interactions with the substrates. PfHT can carry both D-glucose and D-fructose, whereas GLUT1 is selective for D-glucose and GLUT5 for D-fructose. Thus, PfHT can function as a pharmacological target in the development of antimalarials because of its selective affinity for different substrates^74^ ^,^ ^75^. 

In addition, fructose can be used as an energy source in *in vitro* cultures of *P. falciparum*. Concentrations at this stage are lower than those of glucose^76^. 

 The blood phases of the parasite depend on glycolysis for energy production^77^ ^,^ ^78^. Therefore, PfHT is a potential therapeutic target in the exoerythrocytic phase^69^. 

The Lipinski rule is one of the main descriptors used to analyze new prototype compounds for drugs. Thus, the physicochemical profiles of orally administered drugs are mainly based on the Lipinski rule, and it is possible to evaluate the absorption, distribution, metabolism, excretion, and toxicity of the compound, also known as the ADMET property^79^ ^,^ ^80^. The physicochemical characteristics of a compound can be decisive in the success or failure of its biological activity^81^. New antimalarial candidates should exhibit good oral bioavailability and membrane permeability^82^. The SwissADME tool allows determination of the main physicochemical and pharmacokinetic *in silico* properties of compounds^47^. Supplementary Table 1 shows SwissADME profiles of compound 1 and chloroquine.

The partition coefficient (CLogP) is recommended for estimating the toxicological factors^83^. It is known that compounds with high molecular weights and an excessive number of hydrogen acceptors and donor groups have higher difficulty crossing the lipid bilayer of cell membranes^84^.

According to Lipinski’s rule, compound 1 had a CLogP <5 (1.67) and a molecular weight less than 500 g/mol (219.28 g/mol) (Supplementary Table 1). There were three hydrogen acceptor groups that interacted, and no hydrogen donor groups. These data demonstrated that the ADMET properties of compound 1 were acceptable under Lipinski’s rule.

A study by Gleeson^83^ demonstrated that compounds with LogP less than 4 and molecular weight less than 400 g/mol had a promising ADMET profile. Thus, compound 1 also fits the presented criteria and therefore presents the appropriate ADMET properties according to Lipinski and Gleeson^80^ ^,^ ^84^. Compounds with CLogP values ​​less than 3 present a low risk of side effects and toxicity, indicating a low risk of the substance being trapped and stored. When analyzing the toxicological characteristics of compound 1, factors such as mutagenicity, tumogenicity, or irritability were not detected (Supplementary Table 1). In addition, compound 1 has a superior synthetic facility compared to chloroquine, standing out for its solubility in water.

In conclusion, compound 1 has potential as a drug prototype owing to the presentation of appropriate characteristics such as absorption, toxicity, good solubility, low molecular weight, and permeability. These data corroborate the growing interest in synthetic morpholines, as well as the usage diversity of these compounds, as described by Kourounakis^20^, since compound 1 stands out for its antiplasmodial and cytotoxic activity.

## SUPPLEMENTARY MATERIAL

**SUPPLEMENTARY TABLE 1:** Physicochemical properties of compound 1 and chloroquine by SwissADME.

PHYSICOCHEMICAL PROPERTIES	1	CLOROQUINE
Formula	C_13_H_17_NO_2_	C_18_H_26_C_l_N_3_
Molecular weight	219.28 g/mol	319.87 g/mol
Nº, heavy atoms	16	22
Num. heavy atoms	6	10
Fractions Csp3	0.46	0.50
Num. rotate bonds	4	8
Num. H-bond Acceptor	3	2
Num. H-bond Donator	0	1
Molar Refractivity	66,45	97,41
TPSA	36.02 Å²	28.16 Å²
LIPOPHILICITY		
Log *P*_olw_ (ILOGP)	-2.4	3.95
Log *P*_olw_ (XLOGP3)	1.20	4.63
Log *P*_olw_ (WLOGP)	1.21	4.62
Log *P*_olw_ (MLOGP)	-1.05	3.20
Log *P*_olw_ (SILICOS-IT)	2.5	4.32
Consensus Log *P* olw	1.67	4.15
WATER SOLUBILITY		
Log S (ESOL)	-1.97	-4.55
Solubility	2.35e+ mg/ml; 1.07e-02 mol/l	9.05e-03 mg/ml; 2.83e-05 mol/l
Class	Soluble	Moderately soluble
Log S (Ali)	-1.42	-4.95
Solubility	8.40e+00 mg/ml; 3.83e-02 mol/l	3.61e-03 mg/ml; 1.13e-05 mol/l
Class	Soluble	Moderately soluble
Log S (SILICOS-IT)	-3.35	-6.92
Solubility	9.82e-02 mg/ml; 4.48e-04 mol/l	3.86e-05 mg/ml; 1.21e-07 mol/l
Class	Soluble	Poorly soluble
PHARMACOKINETICS		
GI absorption	High	High
BBB *permeant*	Yes	Yes
P-gp substrate	No	No
CYP1A2 inhibitor	No	Yes
CYP2C19 inhibitor	No	No
CYP2C9 inhibitor	No	No
CYP2D6 inhibitor	No	Yes
CYP3A4 inhibitor	Yes	Yes
Log Kp (*skin permeation*)	-6.79 cm/s	-4.96 cm/s
*DRUGLIKENESS*		
Lipinski	Yes; 0 violation	Yes; 0 violation
Ghose	Yes	Yes
Veber	Yes	Yes
Egan	Yes	Yes
Muegge	Yes	Yes
Bioavailability *Score*	0.55	0.55
MEDICINAL CHEMISTRY		
PAINS	0 alert	0 alert
Brenk	0 alert	0 alert
Leandlikeness	No; 1 violation: MW<250	No; 2 violations: Rotors>7, XLOGP3>3.5
Synthetic accessibility	1.54	2.76

## SUPPLEMENTARY MATERIAL

**SYNTHESIS, PURIFICATION, AND STRUCTURAL ELUCIDATION**

The compounds were synthesized according to the classical Mannich reaction using anhydrous ethanol as the solvent and morpholine and piperidine as secondary bases and respective ketones^35^.

**COMPOUNDS**

**Compound 1**

**3-(morpholin-4-yl)-1-phenylpropan-1-one:**

First, 100 mL of anhydrous ethanol, 1.69 g of morpholine hydrochloride (13,66 mmol), 0.41 g paraformaldehyde (13.7 mmol), and 1.64 g acetophenone (13.66 mmol) were stirred under reflux in a sealed vial. The system was turned off after four hours and partitioned three times with water/ethyl acetate. The aqueous phase was collected and dried under vacuum. A small amount of solid residue was crystallized on acetone/ethanol to produce 2.1 g of compound 1 (9.6 mmol of hydrochloride salt) with a yield of 70%.

**Compound 2**

**1-phenyl-3-(piperidin-1-yl)propan-1-one:**

Second, 100 mL of anhydrous ethanol, 1.68 g of piperidine hydrochloride (13.82 mmol), 0.414 g paraformaldehyde (13.8 mmol), and 5.0 g of acetophenone (41.7 mmol) were stirred under reflux in a sealed vial. The system was turned off after three hours and partitioned three times with water/ethyl acetate. The aqueous phase was collected and dried under vacuum. The small solid residue was solubilized in acetone and crystalized overnight at a low temperature (-4 ºC). The crystals were filtered and recrystallized to obtain 3.0 g of compound 2 (11.8 mmol, hydrochloride salt) with a yield of 86%.

**Compound 3**

**2-methyl-3-(morpholin-4-yl)-1-phenylpropan-1-one:**

Third, 100 mL of anhydrous ethanol, 5.5 g of morpholine hydrochloride (44.5 mmol), 1.35 g paraformaldehyde (45 mmol), and 1.5 g propylphenone (11.2 mmol) were stirred under reflux in a sealed vial. The system was turned off after four hours and partitioned three times with water/ethyl acetate. The aqueous phase was collected and dried under vacuum. The small amount of solid residue was crystallized in ethanol at 0 °C to produce 2.0 g of compound 3 (8.6 mmol of hydrochloride salt) with a yield of 76%.

**Compound 4**

**2-methyl-1-phenyl-3-(piperidein-1-yl)propan-1-one:**

Fourth, 100 mL of anhydrous ethanol, 1.37 g of piperidine hydrochloride (11.3 mmol), 0.336 g paraformaldehyde (11.3 mmol), and 4.5 g of propylphenone (33.58 mmol) were stirred under reflux in a sealed vial. The system was turned off after three hours and partitioned three times with water/ethyl acetate. The aqueous phase was collected and dried under vacuum. The small solid residue was solubilized in acetone and crystallized overnight at a low temperature (0 ºC). The crystals were filtered and recrystallized to obtain 2.0 g of compound 4 (7.5 mmol, hydrochloride salt) with a yield of 66%.

**Compounds 5 and 6**

**1-(morpholin-4-yl)decan-3-one and 3-[(morpholin-4-yl)methyl]nonan-2-one:**

For compounds 5 and 6, 100 mL of anhydrous ethanol 5.5 g of morpholine hydrochloride (44.5 mmol), 1.35 g paraformaldehyde (45 mmol), and 2.46 g 2-nonanone (17 mmol) were stirred under reflux in a sealed vial. The system was turned off after four hours and partitioned three times with water/hexane. The aqueous phase was collected and dried under vacuum. The small brown solid was solubilized in acetone/methanol and crystallized at low temperature (0 ºC). The crystals were filtered and recrystallized to obtain a mixture of the two isomeric forms (kinetic and thermodynamic) of compounds 5 and 6 in proportions of 48-52 w/w. As a result, a total yield of 3.85 g (14.0 mmol mixture of hydrochloride salt) was obtained with 82% ketone yield.

**Compounds 7 and 8**

**1-(piperidin-1-yl)decan-3-one and 3[(piperdin-1-yl)methyl]nonan-2-one:**

For compounds 7 and 8, 100 mL of anhydrous ethanol, 1.76 g of piperidine hydrochloride (14.5 mmol), 0.446 g paraformaldehyde (14.5 mmol), and 6.15 g of 2-nonanone (43.3 mmol) were stirred under reflux in a sealed vial. The system was turned off after four hours and partitioned three times with water/ethyl acetate. The aqueous phase was collected and dried under vacuum. The small solid residue was solubilized in ethanol and crystallized overnight at a low temperature (0 ºC). The crystals were filtered and recrystallized to obtain 3.0 g of a mixture of isomers 7 and 8 (10.89 mmol, hydrochloride salt) in 75% yield. High-resolution mass spectrometry (HRMS) (ESI/Q-TOF) m/z: [M+H]+ found for C_15_H_30_NO 240.2322, err = -2.6 ppm.

**PURIFICATION**

The compounds were purified by preparative reverse phase high performance liquid chromatography (RP-HPLC) using a preparative Shim-pack C18 column and Shimadzu HPLC System and CH_3_CN-H_2_O/MeOH- H_2_O as solvents, normal phase (silica gel 60) using hexane/ethyl acetate as solvents, or selective crystallization by partial solubilization and decreasing solvent temperature. TLC using HF254 silica plates (Merck) and MeOH/dichloromethane (DCM) as eluents and spots were visualized under visible light or in a UV chamber to monitor the reactions.

**STRUCTURAL ELUCIDATION**

The compounds were elucidated using high-resolution mass spectrometry (HRMS) and nuclear magnetic resonance (NMR) of 1H and 13C. HRMS was performed using a maXis ETD high-resolution ESI-QTOF mass spectrometer (Bruker) controlled by the Compass1.5 software package (Bruker). Data-dependent fragment spectra were recorded in the collision energy range of 15-60 eV. The ion cooler settings were optimized within a 40-1000 m z-1 range using a calibrant solution of 1 mmol l-1 sodium formate in 50% 2-propanol. Nuclear magnetic resonance (NMR) spectra of 1H and 13C were obtained in a Bruker DRX400 device using deuterium oxide as the solvent and 400 MHz and 100 MHz for 1H and 13C, respectively. Infrared spectra were recorded using an FTIR 8400 Shimadzu spectrophotometer.

**Compound 1**

**3-(morpholin-4-yl)-1-phenylpropan-1-one (AB1)**

^1^H NMR (400 MHz, D_2_O): δ = 3.28-3.33 (m, 4H), 3.63-3.73 (m, 4H), 3.94-3.98 (m, 4H), 7.56-7.62 (m, 2H), 7.71-7.76 (m, 1H), 8.01-8.06 (m, 2H); ^13^C NMR (100 MHz, D_2_O): δ = 43.11, 52.01, 52.15, 63.68, 128.18, 128.92, 134.52, 135.29, 199.59 ppm. HRMS: m/z calcd for C_13_H_18_NO_2_ [M+H]^+^: 220.1337; found: 220.1332.

**Compound 2**

**1-phenyl-3-(piperidin-1-yl)propan-1-one**

^1^H NMR (400 MHz, D_2_O): *δ* = 8.02 (d, 2H, *J =* 8.4 Hz), 7.72 (t, 1H, *J* = 7,4 Hz), 7.58 (t, 2H, *J* = 8.08 Hz), 3.59 (d, 2H, *J* = 11.5 Hz), 3.53 (s, 2H), 3.02 (t, 2H, *J* = 10 Hz), 1.95 (s, 2H), 1.78 (d, 4H, *J* = 12 Hz); ^13^C NMR (100 MHz, D_2_O): *δ* = 199.8, 199.8, 135.3, 134.5, 128.9, 128.1, 53.5, 51.7, 51.6, 22.7, 21.0 ppm. HRMS: *m/z* calcd for C_14_H_20_NO [M+H]^+^: 218.1545; found: 218.1539.

**Compound 3**

**2-methyl-3-(morpholin-4-yl)-1-phenylpropan-1-one**

^1^H NMR (400 MHz, D_2_O): *δ* = 8.06 (d, 2H, *J =* 8.4 Hz), 7.75 (t, 1H, *J* = 7,4 Hz), 7.61 (t, 2H, *J* = 8.1 Hz), 4.26-4.17 (m, 1H), 3.97 (t, 1H, *J* = 5.0 Hz), 3.85 (dd, 3H, *J* = 13.3 Hz, 9.2 Hz), 3.35 (d, 2H, *J* = 3.8 Hz), 3.32 (d, 2H, *J* = 3.9 Hz), 1.31 (d, 3H, *J* = 7.3 Hz); ^13^C NMR (100 MHz, D_2_O): *δ* = 203.4, 134.7, 134.1, 129.1, 128.7, 63.5, 63.4, 58.4, 52.4, 43.1, 36.7, 16.6 ppm. HRMS: *m/z* calcd for C_14_H_20_NO_2_ [M+H]^+^: 234.1494; found: 234.1489.

**Compound 4**

**2-methyl-1-phenyl-3-(piperidein-1-yl)propan-1-one**

^1^H NMR (400 MHz, D_2_O): *δ* = 8.06 (d, 2H, *J =* 8.4 Hz), 7.74 (t, 1H, *J* = 7,5 Hz), 7.60 (t, 2H, *J* = 7.6 Hz), 4.21-4.13 (m, 1H), 3.73 (dd, 1H, *J* = 13.4 Hz, 9.2 Hz), 3.53 (t, 2H, *J* = 15.4 Hz), 3.25 (d, 2H, *J* = 3.8 Hz), 3.21 (d, 2H, *J* = 3.8 Hz), 2.99 (q, 2H, *J* = 12.0 Hz), 1.91 (d, 2H, *J* = 14.4 Hz), 1.49 (t, 1H, *J* = 12.4 Hz), 1.28 (d, 3H, *J* = 7.3 Hz); ^13^C NMR (100 MHz, D_2_O): *δ* = 203.7, 134.7, 134.2, 129.1, 128.7, 57.9, 54.2, 53.7, 44.5, 36.9, 16.7 ppm. HRMS: *m/z* calcd for C_15_H_21_NO [M+H]^+^: 232.1701; found: 232.1696.

**Compounds 5 and 6 (mixture)**

**1-(morpholin-4-yl)decan-3-one and 3-[(morpholin-4-yl)methyl]nonan-2-one**

^1^H NMR (400 MHz, D_2_O): *δ* = 3.69 (dd, 4H, *J =* 13.4 Hz, 9.8 Hz), 3.46 (t, 8H, *J* = 6.9 Hz), 3.24-3.10 (m, 6H), 2.59 (t, 2H, *J* = 7.4 Hz), 2.33 (s, 8H), 1.79-1.55 (m, 4H), 1.29 (s, 16H), 0.87 (s, 6H); ^13^C NMR (100 MHz, D_2_O): *δ* = 213.5, 212.3, 63.6, 63.4, 56.4, 51.9, 51.7, 46.7, 42.3, 35.7, 30.9, 30.7, 29.4, 28.2, 28.1, 28.0, 25.3, 23.0, 21.9, 21.8, 13.3, 13.2 ppm. HRMS: *m/z* calcd for C_14_H_28_NO_2_ [M+H]^+^: 242.2120; found: 242.2115.

**Compounds 7 and 8 (mixture)**

1-(piperidin-1-yl)decan-3-one and 3[(piperdin-1-yl)methyl]nonan-2-one

^1^H NMR (400 MHz, D_2_O): *δ* = 3.61-3.50 (m, 2H), 3.37 (t, 2H, *J* = 6.9 Hz), 3.30-3.24 (m, 10H), 3.13-2.90 (m, 7H), 2.34 (s, 3H), 1.94-1.48 (m, 12H), 1.28 (s, 18H), 0.86 (s, 6H); ^13^C NMR (100 MHz, D_2_O): *δ* = 213.2, 211.3, 56.0, 54.4, 53.4, 51.2, 47.2, 42.4, 36.3, 31.2, 30.9, 29.8, 28.6, 28.5, 28.4, 28.3, 25.5, 23.2, 22.8, 22.6, 22.4, 22.2, 22.0 ppm. HRMS: *m/z* calcd for C_15_H_30_NO [M+H]^+^: 240.2327; found: 240.2322.

**EVALUATION OF MOLECULAR DOCKING OF PHYSICOCHEMICAL AND ADMET PROPERTIES**

All compounds were designed using the MarvinSketch^®^ software program (ChemAxon, Cambridge, MA, USA), and the molecular structures were refined using the MOPAC^®^ software (Stewart Computational Chemistry, Colorado Springs, CO, USA). The compounds were subjected to molecular docking calculations using the AutoDock Vina^®^ program^43^. All calculations were performed using OCTOPUS^®^ program to optimize the virtual screening process. The configuration files were determined using a re-docking step^44^ ^,^ ^45^ ^,^ ^46^. Table 1 lists the molecular targets of the BraMMT.

The physicochemical properties of the eight compounds (1-8) were analyzed using the DataWarrior® software and SwissADME website^47^. Finally, toxicological characteristics of the compounds, such as mutagenicity, tumorigenicity, and irritability, were analyzed using DataWarrior^®^ ^48^.
